# Relational, cognitive, and structural social capital in shaping livelihood resilience in informal agri-food systems

**DOI:** 10.3389/fsoc.2026.1821886

**Published:** 2026-06-02

**Authors:** Darmawan Salman, Nurdahalia Lairing, Nurfadila JS, Ariady Arsal, A. Amidah Amrawaty, Loes Witteveen

**Affiliations:** 1 Social Capital Research Group, Hasanuddin University, Makassar, Indonesia; 2 Graduate School of Hasanuddin University, Makassar, Indonesia; 3 Department of Socio-Economics of Animal Science, Hasanuddin University, Makassar, Indonesia; 4 Research Group of Communication, Participation and Social Ecological Learning (COPSEL), Van Hall Larenstein University of Applied Sciences, Velp, Netherlands

**Keywords:** coordination infrastructure, informal agri-food systems, livelihood resilience, political ecology, risk distribution, social capital

## Abstract

**Introduction:**

Smallholder livelihoods in the Global South operate under persistent uncertainty shaped by price volatility, climate variability, and limited institutional protection. While informal agri-food systems are often framed as inefficient or transitional, their persistence raises questions about how coordination and resilience are sustained in the absence of formal governance structures. This study examines how the relational, cognitive, and structural dimensions of social capital interact to shape livelihood resilience.

**Methods:**

This study draws on qualitative research conducted in Enrekang, Indonesia, using in-depth interviews and field observations involving farmers, traders, and informal financial actors. A multi-actor livelihood analysis was applied to examine how social relations organize everyday economic practices.

**Results:**

The findings show that social capital operates as a coordinating infrastructure linking actors, resources, and practices. Trust-based relationships, shared understandings, and networked arrangements enable adaptive responses such as diversification, informal credit, and network-based market exchanges. These mechanisms sustain livelihood continuity under uncertainty. However, risks are unevenly distributed: farmers remain exposed to production and price risks, while traders and intermediaries absorb market and financial volatility.

**Discussion:**

These findings suggest that resilience reflects the capacity to maintain system function rather than reduce vulnerability. By reconceptualizing social capital as a dynamic and socially embedded coordination mechanism, this study contributes to debates on livelihood resilience and informality, showing that resilience is relationally mediated and shaped by unequal access to social networks and resources.

## 1 Introduction

Smallholder-based agri-food systems in the Global South increasingly operate under persistent and intersecting uncertainties. Price volatility and climate variability are no longer episodic shocks but enduring conditions that shape everyday livelihood decision-making, influencing how farmers and market actors plan, adjust and cope over time (Caviedes et al., 2024; Mohammed et al., 2021). In such contexts, actors must manage not only production risks but also unstable market relations, fluctuating demand, and uneven access to resources, raising fundamental questions about how livelihoods are coordinated and sustained under conditions of structural uncertainty.

Despite this reality, policy and development responses have largely prioritized technical and formal solutions, including productivity enhancement, market integration, and financial inclusion. While important, these interventions often overlook how livelihoods are maintained in the long term. A growing body of literature shows that informal agri-food systems remain central to agricultural production and exchange in several rural economies in developing countries. In these systems, transactions occur without formal contracts, coordination relies on trust and reputation, and access to capital is mediated through personal networks rather than formal financial institutions (Rouillé et al., 2024; Song, 2024). Thus, informality operates as a mode of economic coordination under conditions of weak governance and limited institutional protection (Caviedes et al., 2025).

Livelihood studies have provided valuable insights into how rural households combine activities and assets to cope with risk. However, while this literature richly documents what actors do, it offers more limited explanations of how livelihood practices are coordinated and sustained over time within uncertain systems. Recent work on livelihood resilience addresses this gap by conceptualizing resilience as the capacity to absorb disturbances, adapt to change, and sustain core functions under conditions of uncertainty (Olsson et al., 2004; Scoones, 1998). Importantly, resilience is increasingly understood as an emergent property shaped by institutional arrangements, power relations, and everyday practices rather than as a purely technical outcome (Boillat and Speranza, 2023; Ruben, 2024). Recent empirical work shows that livelihood strategies are shaped by how actors mobilize and recombine available assets in response to risk (Irawan, 2023).

Within this debate, social capital has gained prominence as a key factor in shaping adaptation and resilience. Trust-based relationships, shared norms, and social networks enable access to information, resources, and collective action, particularly in areas where formal institutions are lacking (Ajates, 2021; De Grandpré et al., 2022). However, social capital remains analytically underdeveloped in livelihood research. It is often treated as a static asset or community attribute rather than a dynamic set of social relations through which coordination, risk sharing, and decision-making are continuously negotiated. Moreover, existing studies frequently focus on single actor groups, such as farmer organizations, without adequately examining how multiple actors—farmers, traders, laborers, and informal financial agents—are interconnected within shared livelihood systems.

Recent studies have applied a multidimensional conceptualization of social capital in various empirical contexts. For instance, research on supply chain relationships shows how structural and relational dimensions shape coordination and exchange dynamics (Singh et al., 2023), whereas studies on micro-enterprise performance highlight the interaction between cognitive and relational capital in influencing economic outcomes (Al Muniady et al., 2015). Similarly, research on digital communities demonstrates how structural, cognitive, and relational dimensions shape user engagement and network participation (Kim et al., 2021). Despite these contributions, existing studies have largely examined these dimensions in organizational, enterprise or digital settings. However, their combined role in shaping livelihood strategies within informal agri-food systems remains insufficiently elaborated. This gap is significant because social capital is a key component of livelihood assets, and its multidimensional interaction is central to understanding how coordination, risk distribution, and adaptation occur in practice. Accordingly, this study aims to examine how the relational, cognitive, and structural dimensions of social capital interact to shape livelihood resilience among smallholder households in informal agri-food systems.

Consequently, there is limited empirical understanding of how multidimensional social capital operates as a coordinating mechanism in everyday livelihood practices, particularly in contexts where institutional protection remains weak and informal arrangements substitute for formal governance. Accordingly, this study examines how the relational, cognitive, and structural dimensions of social capital interact to coordinate economic activities, distribute risk, and shape livelihood resilience within informal agri-food systems.

Social capital and livelihood resilience are conceptualized as interrelated and processual phenomena that shape the coordination and sustainability of livelihoods under persistent uncertainty. Social capital is understood as a multidimensional construct comprising relational, cognitive, and structural dimensions (Nahapiet and Ghoshal, 1998), through which social relations enable coordination and access to resources embedded in livelihood systems. While the analysis foregrounds social capital, it treats social relations as mediating and actively shaping the production, access, and use of other livelihood capitals, including infrastructure, technologies, land, labor, finance, and knowledge. Building on this conceptualization, the analysis adopts a multidimensional perspective that examines how these dimensions interact as socially embedded processes rather than as separate analytical variables. By shifting attention from resilience outcomes to the social processes that stabilize everyday exchanges, this study shows how informal arrangements enable continuity while shaping risk management and distribution across actors.

This study makes three contributions to extant literature. First, it reconceptualizes social capital as a coordinating livelihood infrastructure that organizes economic exchanges in the absence of institutions. Second, it demonstrates how interactions between the relational, cognitive, and structural dimensions shape both adaptation and the distribution of risk across the actors. Third, by distinguishing coordination from protection, it shows how resilience can coexist with persistent vulnerability, thereby challenging interpretations that equate adaptability with reduced precariousness.

These dynamics were empirically examined using a qualitative research design that enabled close engagement with everyday practices and social relations. The following section outlines the study context, data collection, and analytical procedures.

## 2 Literature review

The study of livelihood resilience in informal agri-food systems requires a conceptual framework that captures both the relational foundations of social organization and the institutional conditions under which economic activities are coordinated. Existing scholarship on social capital and informal agri-food systems provides important insights into these dynamics, particularly through the development of multidimensional approaches to social capital and their application in diverse empirical contexts.

### 2.1 Social capital and livelihood system

Social capital is commonly understood as the resources embedded in social relations that can be accessed and mobilized through networks of interactions (Lin, 2002). Foundational perspectives conceptualize social capital as arising from social structures that facilitate action and provide access to resources (Bourdieu, 2008; Coleman, 1988). Building on these contributions, a multidimensional framework distinguishes the structural, cognitive, and relational dimensions of social capital (Nahapiet and Ghoshal, 1998). The structural dimension refers to network configurations and patterns of interaction, the cognitive dimension captures shared meanings, norms, and interpretive frameworks, and the relational dimension reflects trust, reciprocity, and the quality of social relationships.

The multidimensional conceptualization emerged in response to earlier approaches that treated social capital as either a network structure or shared norms and trust. By integrating these dimensions, the framework offers a more comprehensive analytical lens for examining how social relations shape coordination, meaning-making, and institutionalized interaction patterns (Nahapiet and Ghoshal, 1998). In contrast to form-based approaches, such as bonding, bridging, and linking social capital, which categorize types of relationships, the multidimensional perspective emphasizes the simultaneous and interactive functioning of different relational elements.

Simultaneously, form-based approaches continue to provide important insights into how different types of relationships operate in specific contexts. Recent studies have demonstrated that bonding, bridging, and linking social capital play differentiated yet complementary roles across the risk and response phases. For instance, bonding ties often dominate during the onset of a crisis, enabling immediate protection and mobilization within households and close-knit networks. Bridging ties become more prominent during periods of disruption by facilitating access to support beyond immediate circles, whereas linking ties are particularly critical during recovery phases by connecting actors to institutional resources and external support systems. Importantly, these forms do not operate independently; they interact and reinforce one another over time, shaping adaptive capacity through their combined effects.

Empirical applications of this framework have expanded across organizational, inter-firm, and digital contexts. Evidence shows that structural, cognitive, and relational dimensions jointly influence coordination, knowledge exchange, and performance outcomes (Kim et al., 2021; Al Muniady et al., 2015; Singh et al., 2023). These studies demonstrate that analyzing social capital as a single variable obscures the mechanisms through which social relations shape economic outcomes, highlighting the importance of examining the interactions between dimensions of social capital.

Within livelihood systems, social capital plays a critical role in shaping adaptive capacity by enabling access to financial, human, natural, and physical resources (Donoghue and Sturtevant, 2007; Wuepper et al., 2018). In line with the sustainable livelihood framework, adaptation strategies emerge as responses to livelihood risks and are shaped by the availability and interaction of these assets (Ellis and Freeman, 2004; Irawan, 2023; Scoones, 1998). In this context, social capital supports information exchange, collective action, and coordination among actors engaged in livelihood activities (Aldrich and Meyer, 2015; Cinner et al., 2018). In resource-constrained and hazard-prone environments, it becomes particularly significant in compensating for limitations in formal institutions and sustaining resilience processes (Hudson et al., 2020).

Despite these contributions, much of the livelihood literature continues to treat social capital primarily as an enabling asset rather than as a process through which coordination is actively organized and maintained. Consequently, limited attention has been paid to how structural, cognitive, and relational dimensions interact to shape livelihood practices across multiple actors within a shared system. This limitation is particularly relevant in informal agri-food systems, where coordination is embedded in everyday social relations rather than formal institutional arrangements.

### 2.2 Informal agri-food system and social capital

Informal agri-food systems are characterized by socially embedded exchanges in which transactions, coordination, and resource flows are governed by trust-based relationships rather than formal contracts and institutions (Woolcock and Narayan, 2000). Market interactions are mediated through personal networks involving farmers, traders, laborers, and financial actors, reflecting the central role of social relations in organizing economic activity (Mohan and Mohan, 2002). Empirical studies in rural and agricultural contexts show that informal agri-food systems rely heavily on social capital to sustain production, distribution, and recovery processes (Azad and Pritchard, 2023). Bonding ties facilitate access to labor and mutual support, bridging networks enable information exchange and market access, and linking relationships connect actors to external institutions (D. P. Aldrich and Meyer, 2015; Hawkins and Maurer, 2010; Nguyen-Trung et al., 2020). This underscores the importance of social capital in supporting livelihood resilience, particularly in contexts characterized by weak governance and limited institutional support.

However, existing research in rural and agricultural settings has largely adopted form-based or single-dimensional approaches to social capital research. While these approaches provide useful categorizations of social relationships, they are less effective in capturing the interaction of different dimensions of social capital. The application of a multidimensional framework in these contexts remains limited, particularly in examining how structural, cognitive, and relational dimensions jointly shape everyday coordination and livelihood resilience.

This gap is significant because rural livelihood systems are inherently multi-actor and depend on overlapping social relations that cannot be adequately captured using isolated or categorical approaches. Therefore, understanding how these dimensions interact is critical for explaining how coordination, risk distribution, and adaptation are sustained within informal agri-food systems.

## 3 Methods

### 3.1 Study context

This study was conducted in the highland area of Enrekang District, Indonesia, a predominantly agrarian region where horticultural production is a central component of local livelihoods. Agricultural activities are organized through smallholder farming systems focused on perishable commodities, with production managed at the household level and supported by a combination of family labor and seasonal work.

A key institutional feature of the area is the Agribusiness Sub-Terminal *(STA/Sub-Terminal Agribisnis)* in Enrekang, which functions as the primary market hub for agricultural products in the region. The STA provides a physical and relational space where farmers, traders, and intermediaries regularly interact, facilitating product aggregation, exchange, and supply chain coordination. Beyond its role as a marketplace, the STA serves as a focal site where social relationships are formed, maintained, and enacted.

The analytical relevance of Enrekang lies in the dominance of informal coordination mechanisms within the agri-food system. Field observations indicate that key economic activities, including price formation, credit provision, labour recruitment, and market access, are largely governed by trust-based relationships, informal agreements, and repeated interactions rather than formal contracts or institutional arrangements. This context provides a critical empirical setting in which the relational, cognitive, and structural dimensions of social capital are not only present but are also actively intertwined in shaping everyday livelihood practices in the region. Thus, Enrekang offers a theoretically relevant case for examining how multidimensional social capital operates as coordinating infrastructure in informal agri-food systems. This makes the case particularly suitable for examining social capital not as a static resource but as a dynamic process embedded in everyday economic coordination.

### 3.2 Research design

This study adopted an exploratory qualitative research approach to capture the complexities of livelihood practices and social relations within informal agri-food systems. A qualitative design is particularly suited for examining relational, context-specific, and often tacit processes that cannot be captured meaningfully through standardized indicators or survey-based approaches.

This study was guided by a multi-actor livelihood analysis. Rather than focusing on a single group, this study examines the interactions among multiple actors involved in the production, circulation, and financing of agricultural commodities in the region. This approach recognizes that livelihood resilience is not produced by farmers alone but emerges from the interconnected relationships among farmers, traders, laborers, and informal financial actors.

The choice of a multi-actor perspective is justified by the nature of this research question. Informal agri-food systems operate through dense social networks that cross occupational and institutional boundaries. Understanding how livelihood resilience is produced requires attention to the relational dynamics that link different actors and their roles within shared livelihood systems. Examining only one category of actors provides an incomplete and potentially misleading picture of how coordination and adaptation occur.

### 3.3 Data collection

Data were collected through in-depth interviews and field observations during multiple field visits. Using a purposive multi-actor sampling strategy, this study engaged 26 informants representing diverse positions within the informal horticultural livelihood system. The selection criteria emphasized functional roles across production, trade, financial intermediation, and institutional coordination while ensuring variations in gender and network position of the selected respondents (see Table 1).

**Table 1:** Categories of informants and their roles within the informal agri-food system.

Category of informant	Number	Gender composition	Position within the livelihood system
Smallholder farmers	5	Male	Primary producers; crop and marketing decision-makers
Women farmers/KWT members	5	Female	Production, savings management, household income stabilization
Farmer group leaders	2	Male and female	Organizational coordination and linkage to formal programs
Agricultural laborers	2	Female	Seasonal labor and knowledge transmission
Collecting traders	3	Male and female	Aggregation and provision of input-based credit
Intermediary traders	3	Male and female	Regional distribution and price mediation
STA managers	1	Male	Market infrastructure and transaction coordination
Arisan group managers/KWT member	2	Female	Community-based rotating savings schemes
Local financial service agent	1	Female	Micro-financial intermediation, Informal credit and liquidity circulation
District government agricultural officers	2	Male and Female	Formal extension, regulatory oversight, and program implementation

Observational data complemented the interview material, providing context for everyday interactions. Observations have focused on market exchanges, including price negotiations, product handling, and farmer–trader interactions. Group meetings, such as women’s group gatherings or informal collective discussions, were observed to understand the organizational practices and social dynamics. Farming practices and work routines in the fields were also observed to capture how production activities were organized and how labor was coordinated in the village. Together, these data sources enabled a grounded and nuanced understanding of how informal social relations and organizational arrangements shape livelihood resilience in this study’s context.

### 3.4 Data analysis

Data were analyzed using an iterative thematic approach guided by the study’s conceptual framework, as follows. The analysis began with open coding of the interview transcripts and field notes, focusing on everyday livelihood practices and forms of coordination among actors within the informal agri-food system. This initial coding stage prioritized the participants’ accounts of concrete actions, interactions, and decision-making processes, allowing analytical categories to emerge inductively from the data set.

The initial codes were organized into three analytical themes corresponding to the relational, cognitive, and structural dimensions of social capital, which enabled the analysis to examine how social relationships, shared understandings, and organizational arrangements shape the enactment of livelihood strategies within a broader livelihood system. Rather than treating these dimensions as predefined variables, they were used as sensitizing concepts to trace how coordination and cooperation were enacted across livelihood practices in the study.

Cross-actor analysis was conducted to identify recurring patterns and variations in the coordination of livelihood practices among farmers, traders, and other market participants. This analytical step allowed for a comparison across actor groups, highlighting how similar social relations and organizational arrangements were experienced and mobilized differently depending on actors’ positions within the livelihood system. Through this process, livelihood resilience was interpreted as an emergent outcome of the interactions between the social capital dimensions and livelihood system conditions.

Throughout the analysis, attention was paid to the iterative movement between empirical material and conceptual interpretation, enabling theoretical refinement based on observed practice. This study seeks analytical rather than statistical generalization, contributing to a process-oriented understanding of how livelihood resilience is produced in informal agri-food systems in developing countries.

### 3.5 Analytical framework

This study adopts an analytical framework that integrates social capital theory (Nahapiet and Ghoshal, 1998) with a livelihood-system perspective (Scoones, 1998). The Sustainable Livelihoods Framework conceptualizes livelihoods as shaped by interactions among multiple forms of capital within a broader vulnerability context and an institutional environment. Building on this perspective, the present study analytically foregrounds social capital as the primary mechanism through which everyday livelihood practices are coordinated, situating it within a wider configuration of livelihood assets and structural conditions.

Social capital is conceptualized using a multidimensional framework that distinguishes between relational, cognitive, and structural dimensions of social capital. Rather than isolating social capital from other livelihood capitals, this study positions it as a coordinating mechanism that co-produces and co-organizes the access, utilization, and material expression of human, financial, natural, and physical capitals. In this sense, social capital does not substitute other forms of capital but conditions how they are mobilized, combined, and sustained in everyday livelihood practices. This approach emphasizes the role of socially embedded processes, which is particularly relevant in informal agri-food systems, where economic activities are not governed by formal institutions but are sustained through repeated interactions, shared understanding and networked relationships.

Relational social capital refers to the quality of relationships among actors, including trust, reciprocity, reputation, and repeated interactions, which enable coordination in uncertain conditions. Cognitive social capital captures the shared knowledge, norms, and interpretive frameworks that shape how actors understand risks, opportunities, and acceptable practices. Structural social capital refers to network configurations and organizational arrangements that provide the context in which social relations are enacted and reproduced. The results include both informal networks and semi-formal market institutions, particularly the Agribusiness Sub-Terminal (STA), which functions as a key coordination hub connecting farmers, traders and intermediaries.

Within this framework, livelihood resilience is understood as an emergent outcome of the interaction between these dimensions rather than the result of individual strategies alone. The effectiveness of social capital is conditioned by access to other livelihood resources, such as land, labor, finance, and infrastructure; however, it plays a central role in coordinating how these resources are accessed and utilized. This positioning extends the Sustainable Livelihoods perspective by examining how social capital operates as a coordinating infrastructure within informal agri-food systems.

**Figure 1:**
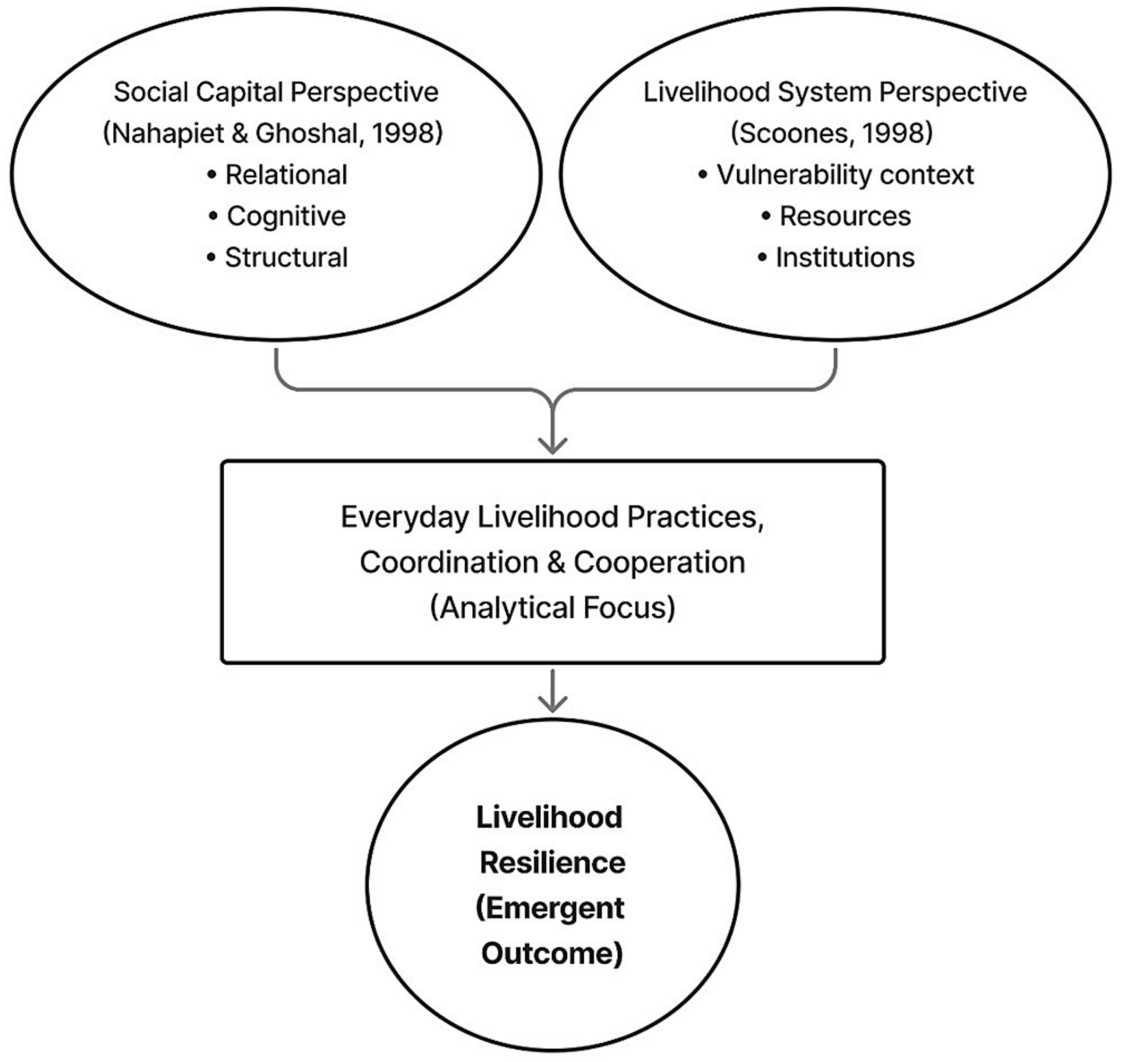
Analytical framework integrating social capital and livelihood system perspectives to examine how everyday livelihood practices mediate the emergence of livelihood resilience. Source: author’s elaboration.

The analytical framework presented in Figure 1 synthesizes these relationships and guides the organization and interpretation of empirical findings.

### 3.6 Ethical considerations

The research protocol was reviewed and approved by the Institutional Review Board of Hasanuddin University (Approval No. 02231/UN4.22.2/PT.01.04/2025, dated June 3, 2025). Prior to the fieldwork, formal permission to conduct the study was obtained from the relevant local authorities at the study site (Approval No. 73.16/1490/DPMPTSP/ENR/IP/VI/2025, dated July 25, 2025).

This study was conducted in accordance with the established ethical principles of social research involving human participants. All participants were informed of the purpose and scope of the study and provided informed consent prior to their participation. To ensure confidentiality, personal identifiers were removed from all transcripts and pseudonyms were used to report qualitative data.

Data collection involved non-invasive qualitative methods, including in-depth interviews, focus group discussions, and field observations of adult participants engaged in agricultural production, market activities and local governance. The researchers remained attentive to their positionality during fieldwork and analysis, recognizing how prior experience and social interactions in the study context could shape the data interpretation.

## 4 Results

This section presents the empirical findings of the study, focusing on how livelihood activities are coordinated through relational, cognitive, and structural dimensions of social capital within the agri-food system in Enrekang.

The analysis is based on interview accounts and field observations of farmers, traders, agricultural laborers, women’s groups, and informal financial actors operating within a horticulture-based production system. Rather than treating social capital as a static asset, the findings are organized to show how coordination emerges in everyday practices under conditions of price volatility, production risk, and limited institutional support. The section documents observable patterns of interaction through which actors organize production, exchange, and access to resources.

The presentation remains grounded in empirical accounts without introducing theoretical interpretation. Analytical interpretation is developed in the Discussion.

### 4.1 Relational social capital: trust-based coordination in everyday livelihood practices

Relationships between farmers and traders form the backbone of local livelihood practices. Most farmers sell their produce through long-term trading relationships with specific traders. These relationships are maintained through a system of *langganan*, in which transactions are repeated over multiple production cycles. In many cases, traders provide agricultural inputs in advance, with payments deferred until harvest.

*“I only work with farmers I already know, because without trust I would not provide inputs in advance”* (Trader, male, 45).

*“We keep selling to the same trader because they always buy our harvest, even when prices are low.”* (Farmer, male, 52).

Trust in these relationships is selective and closely linked to reputation. Traders report that they only extend advanced inputs or flexible payment arrangements to farmers whose production practices and repayment behavior they know well. Similarly, farmers indicate that they prefer traders who consistently purchase their harvest, provide timely payments, and apply prices that are considered reasonable under prevailing market conditions. Decisions to maintain or terminate trading relationships are often based on past experiences of reliability rather than short-term price advantages.

The distribution of risk within these arrangements varies across actors. Field accounts indicate that traders are exposed to credit and repayment risks due to advance provision of inputs, while farmers face greater uncertainty related to price volatility, production outcomes, and fluctuating market conditions.

*“Sometimes we give inputs first, but payment comes late or not fully—that is the risk we take”* (Trader, male, 50).

Relational social capital also structures labor arrangements. Although collective labor practices have declined, labor recruitment remains embedded in personal networks and informal coordination mechanisms.

*“During the dry season, my own farm cannot be cultivated, so I look for other work as a laborer on neighboring or relatives’ farms”* (Agricultural laborer, female, 36).

*“There is usually a coordinator who organizes the workers; the landowner contacts this coordinator when they need labor”* (Agricultural laborer, male, 42).

*“We usually call workers we already know or those recommended by friends. It’s easier because we trust how they work*.” (Farmer, female, 41).

These relational arrangements also underpin the collective organization of infrastructure development. Field evidence shows that farmer groups mobilize trusted networks to coordinate labor contributions in the construction of farm access roads, initially developed through *gotong royong* and later supported by government programs. As one farmer explained:

*“We worked together to open the farm road, and later the government supported it with concrete so cars could access the area and make it easier to transport inputs and harvests”* (Farmer group leader, male, 75).

Moral considerations further shape interactions. Values such as honesty, commitment, and maintaining a “good name” influence partner selection and decision-making. Overall, relational social capital is enacted through repeated interactions and reputation-based arrangements that sustain coordination while remaining contingent on ongoing trust and performance.

### 4.2 Cognitive social capital: shared knowledge and local meaning-making in livelihood practices

Cognitive social capital is manifested through shared knowledge, practical reasoning, and locally grounded understanding that guide livelihood decisions. Knowledge is largely experiential, context-specific, and socially validated rather than formally codified. Farmers report that most production decisions are grounded in accumulated experience and direct observation. Knowledge is acquired through practice, family transmission, and interaction with other farmers.

*“If we get new information, we don’t apply it directly; we try it first on a small plot, and if it works, then we continue”* (Farmer, male, 47).

*“We believe what we see in the field—if it doesn’t work here, we don’t follow it”* (Farmer, female, 39).

This pattern was also observed by an extension officer:

*“Farmers usually do not immediately adopt new recommendations; they prefer to test them first and only continue if the results are visible in their own fields”* (Extension officer, female, 58).

Economic considerations further shape adoption decisions:

*“Farmers tend to adopt good agricultural practices when there are clear economic benefits …”* (Extension officer, male, 40).

New information from extension services, traders, or digital platforms is treated as provisional and must be validated through practice. This reflects a shared cognitive framework in which knowledge gains legitimacy through observable outcomes rather than formal authority.

Shared experiential knowledge also informs technological adaptation within farmer networks. Farmers describe how irrigation practices, including the development and adjustment of sprinkler systems, are collectively refined based on local conditions and repeated experimentation. As one farmer noted:

*“We developed our own sprinkler system together, adjusting it based on what works for our land and crops”* (Farmer, male, 52).

Digital infrastructure further supports knowledge exchange. Farmers use mobile phones and social media platforms to access information and communicate with others.

*“We often look for information through social media or ask others through our phones when we need to know something about farming”* (Farmer, male, 45).

*“I am not a member of a farmer group; I learned how to use a pH tester from YouTube”* (Farmer, male, 30).

Shared meanings regarding risk and livelihood priorities are also evident. Price fluctuations, climate variability, and simultaneous harvests are widely understood as normal conditions rather than exceptional events.

*“Coffee is like savings for us. We keep it for when we really need money.”* (Farmer, male, 55).

These shared interpretations shape diversification strategies, risk tolerance, and decision-making across production cycles. Overall, cognitive social capital operates through collectively recognized knowledge practices and shared meanings that guide how actors interpret uncertainty and make livelihood decisions.

### 4.3 Structural social capital: informal institutions and organisational arrangements

Structural social capital is reflected in informal institutions and organizational arrangements that enable repeated interactions among actors. These structures provide continuity in livelihood practices despite limited formal institutional support.

Formal farmer groups exist but play a limited role in everyday practices.

*“The farmer group is mostly for government programs; we rarely use it to discuss farming problems”* (Farmer, male, 54).

*“Group activities are usually active when there are programs…”* (Farmer group leader, male, 50).

A district agricultural officer explained:

*“Our current priority is to strengthen food security…”* (Government officer, male, 48).

These accounts indicate that formal farmer groups are primarily activated through externally driven programs.

In contrast, women’s groups (KWT) exhibit more consistent organizational practices embedded in routine activities.

*“In the women’s group, we meet regularly, save together, lend to each other, and run small businesses”* (KWT member, female, 42).

Among traders, informal organizational structures are highly developed.

*“We have our own system… and we also have arisan, so money keeps moving”* (Trader, male, 50).

An *arisan* group manager—a locally organized rotating savings arrangement—explained:

*“We organize savings rotation so members can access cash when needed…”* (Arisan manager, female, 45).

Informal financial arrangements extend beyond trader networks.

*“If we need money quickly, we borrow from people we trust or use BRIlink…”* (Farmer, male, 44).

*“People come to us because transactions are faster…”* (Financial agent, female, 37).

Structural coordination is also evident in the operation of market facilities such as the Sub Terminal Agribusiness (STA), which functions as a central node linking farmers and traders across locations. As the STA manager explained:

*“This market connects farmers and traders from different areas, and most transactions depend on direct negotiation and existing relationships”* (STA manager, male, 57).

Farmers and traders use this facility to coordinate sales, access buyers, and manage the flow of goods across markets. Across these accounts, structural social capital is expressed through organizational arrangements sustained by ongoing interaction and embedded in everyday livelihood practices.

### 4.4 Coordinated livelihood practices and resilience outcomes

The findings show how coordination is expressed in everyday livelihood practices across production, exchange, and resource access. These practices are structured through ongoing interaction patterns among actors, shaping how infrastructure, technologies, and market arrangements are developed and used within the system. Across the cases presented, coordination is not only reflected in social relations but is also materialized through collective practices that underpin the development of infrastructure and technological arrangements.

Collective action and network-based relational arrangements underpin the development and use of shared infrastructure. The construction of farm access roads, for example, emerges from coordinated labor mobilization within farmer groups and local networks, enabling improved access to production areas and facilitating the movement of inputs and harvested products. Similarly, the agro-market (STA) functions as a key organizing platform where actors aggregate products, negotiate transactions, and manage distribution across locations. These arrangements illustrate how infrastructure is not externally imposed but co-produced through repeated interaction among actors.

Technological practices are also shaped through these interaction patterns. Irrigation systems, such as locally developed sprinkler technologies, are adapted through shared learning and experimentation, where farmers collectively adjust practices based on observed outcomes. In this context, technology use is embedded in relational dynamics rather than driven solely by external technical recommendations.

Digital tools further extend these collective practices. The use of mobile phones and social media platforms enables actors to access information, communicate with trading partners, and organize transactions without relying on formal market channels. These coordinated practices contribute to the capacity of actors to sustain livelihood activities under persistent uncertainty. Rather than reflecting the absence of risk, livelihood resilience is expressed through the continuity of economic practices across fluctuating market conditions and changing production environments.

A first observable outcome is income continuity. Farmers and traders describe how ongoing relationships and flexible arrangements enable transactions to continue even when prices are unstable or production outcomes are uncertain. As one farmer explained:

*“Even when prices go down, we still sell to the same trader because we need to keep the cash flowing”* (Farmer, male, 52 years).

Similarly, traders maintain purchasing activities despite uncertainty in downstream markets, relying on established relationships to sustain the flow of goods. These practices indicate that continuity is maintained through relational arrangements rather than formal guarantees.

A second outcome is adaptive flexibility, reflected in the ability of actors to adjust their strategies in response to changing conditions. Farmers diversify crops, shift between commodities, and adapt production practices based on observed outcomes and market signals. Traders similarly adjust sourcing strategies, modify trading routes, and respond to price variations across markets. These adjustments emerge through ongoing interaction and information exchange rather than centralized planning.

A third outcome is recovery capacity, expressed in the ability to absorb and respond to disruptions. Informal credit arrangements, savings practices, and support from social networks enable actors to continue operating during periods of financial constraint or production loss. As one trader noted:

*“If farmers cannot pay immediately, we wait or adjust—it’s part of maintaining the relationship”* (Trader, male, 50).

These arrangements allow actors to manage short-term shocks, although they do not eliminate longer-term exposure to risk.

A fourth outcome is continued livelihood participation, where actors remain engaged in production, trade, and related activities despite persistent uncertainty. Farmers continue cultivating land even when returns are uncertain, while traders and laborers sustain their roles within the system through continuous coordination. Participation is therefore maintained not because conditions are stable, but because social arrangements enable actors to remain connected to livelihood opportunities.

Across these outcomes, risk is not evenly distributed. Farmers remain exposed to production uncertainty, input costs, and price fluctuations at the farm gate, while traders and intermediaries absorb risks related to market volatility, delayed payments, and capital turnover. These patterns indicate that resilience is associated with the capacity to sustain participation and ongoing economic coordination, while the distribution of risk varies across actors depending on their position within the system.

These outcomes are not shaped by social capital alone. Their realization depends on interactions with other livelihood capitals, including access to land and natural resources, availability of labor and skills, financial resources, and supporting infrastructure. Socially embedded coordination processes enable actors to mobilize these capitals in practice, but the capacity to sustain livelihood activities remains contingent on their availability and distribution across actors.

Overall, livelihood resilience in this context is expressed through continuity, adaptability, and participation in economic activities supported by these socially embedded coordination mechanisms. These findings provide an empirical basis for understanding resilience as an emergent property of everyday livelihood practices, rather than a condition of reduced vulnerability.

## 5 Discussion

This section situates the empirical findings within the broader debates on livelihood resilience, social capital, and informality in agri-food systems in the Global South. Building on these results, the discussion moves beyond descriptive patterns to examine how everyday livelihood practices are coordinated and sustained under conditions of persistent price volatility, climate variability and weak institutional protection. This study aligns with recent scholarship that conceptualizes uncertainty as a structural condition that shapes agri-food systems and livelihood trajectories (Caviedes et al., 2025; del Mar Eva Alemany et al., 2021). Within this context, the discussion advances the argument that social capital operates not merely as a supplementary resource but also as a core infrastructure through which informal agri-food systems maintain coordination, manage risks, and generate livelihood resilience.

Building on the integrated findings presented in Section 4, this study further emphasizes that coordination processes are not only social but also material, shaping the development and use of infrastructure, technologies, and market arrangements within informal agri-food systems.

### 5.1 Social capital as livelihood coordination infrastructure

This study reconceptualizes social capital as a coordination infrastructure that organizes livelihood practices in informal agri-food systems. Drawing on the multidimensional framework of relational, cognitive, and structural social capital (Nahapiet and Ghoshal, 1998), the findings demonstrate that these dimensions operate not as discrete analytical categories, but as interdependent processes that shape how actors coordinate production, exchange, and resource mobilization.

The findings further show that these dimensions interact in practice, where processes across relational, cognitive, and structural forms of social capital materialize not only in social interaction but also in the development and use of infrastructure, technologies, and market arrangements. Rather than functioning as a static asset, social capital operates as a dynamic mechanism through which actors mobilize and combine different livelihood resources in response to changing conditions.

Importantly, the findings show that these processes extend beyond social interaction to the material organization of livelihood systems. Infrastructure, technologies, and market facilities—often categorized as physical capital—are not externally given inputs, but are co-produced through the interaction of relational, cognitive, and structural social capital.

This perspective contributes to ongoing debates in livelihood studies, which have traditionally framed social capital as a component of a broader portfolio of assets (Ellis and Freeman, 2004; Scoones, 1998). While this framing has been useful for understanding asset-based strategies, it tends to treat social capital as parallel to other capitals, such as financial, human, natural, and physical resources. The findings from Enrekang suggest a different model configuration. Social capital does not operate independently of these forms of capital; instead, it mediates their access, combination, and sustainability in practice. Trust-based credit arrangements shape access to financial capital, while labor networks mobilize human resources. At the same time, experiential knowledge guides how natural resources are used and adapted in practice. In this sense, social capital functions less as a discrete asset and more as a relational infrastructure that organizes the use of other livelihood capitals. This also suggests that the relationship between social capital and other livelihood capitals is not parallel but generative, where socially embedded processes actively shape how material and economic resources are produced, accessed, and sustained in practice.

This reconceptualization also extends the multidimensional approach to social capital, which distinguishes between relational, cognitive, and structural dimensions (Nahapiet and Ghoshal, 1998). Previous studies have applied this framework primarily in organizational and market settings, including supply chain relationships (Singh et al., 2023), micro-enterprise performance (Al Muniady et al., 2015), and digital communities (Kim et al., 2021), demonstrating how social capital operates through the interaction of network structures, shared meanings, and relationship quality. However, these applications have largely focused on formal or semi-formal contexts. In contrast, this study extends the framework by showing how these dimensions operate as livelihood coordination infrastructure across multiple actors within informal agri-food systems under conditions of structural uncertainty.

Empirical findings from Enrekang clearly illustrate this interaction: relational social capital, expressed through trust-based relationships between farmers, traders, and laborers, enables transactions, credit provision, and labor coordination to continue despite uncertainty. This aligns with the literature on relational governance in informal markets, which highlights the role of trust in sustaining exchanges under weak formal institutions (Milone and Ventura, 2024; Moreno-Miranda and Dries, 2024). Simultaneously, the findings reinforce critiques cautioning against idealizing trust-based systems. Trust facilitates coordination but does not eliminate risk; instead, it redistributes exposure across actors, often placing greater financial risk on traders while allowing farmers to maintain production continuity (Johnson et al., 2023; Mkhize and Cele, 2025).

Cognitive social capital complements these relational dynamics by providing a shared frame of meaning through which actors interpret uncertainty and evaluate viable strategies. Farmers and traders in Enrekang do not orient their decisions toward profit maximization alone but prioritize income continuity, risk spreading, and stable livelihood. This supports critiques of conventional livelihood strategy literature, which often reduces decision-making to economic rationality (Pagnani et al., 2021; Ruben, 2024). Instead, decision-making is shaped by socially shared understandings of acceptable risk, sufficiency, and long-term sustainability. These shared cognitive frames align individual actions with broader system-level continuity, allowing coordination to emerge without formal planning or centralized control.

Structural social capital anchors relational and cognitive processes within routine organizational arrangements. Informal institutions, including women’s groups (KWT), trader networks, *arisan* systems, BRIlink services, and agro-market facilities such as the STA provide stable arenas for repeated interaction, resource circulation, and coordination. These findings resonate with studies describing “nested markets” and hybrid institutional arrangements, where economic exchange is embedded within social relations and informal rules (Milone and Ventura, 2024; Stoeva et al., 2024). Simultaneously, the findings challenge the policy assumption that formal farmer organizations are the primary vehicles of coordination. In Enrekang, formal groups are often activated around program implementation, whereas informal arrangements operate continuously and are more closely aligned with everyday livelihood needs.

Taken together, the interaction between the relational, cognitive, and structural dimensions of social capital enables informal agri-food systems to function as coordinated and durable livelihood systems rather than as fragmented or transitional market forms. This supports recent arguments that treating social capital as a single variable obscures the mechanisms through which coordination and resilience emerge (Huang et al., 2024). By conceptualizing social capital as a coordination infrastructure embedded within livelihood systems, this study clarifies how informal arrangements sustain production, exchange, and recovery under conditions of persistent uncertainty, while simultaneously revealing how risks and opportunities are unevenly distributed across actors.

### 5.2 Resilience through informality: strengths and trade-offs

These findings reinforce a growing body of scholarship that conceptualizes informal agri-food systems as durable and adaptive livelihood arrangements rather than residual or transitional market forms (Milone and Ventura, 2024; Moreno-Miranda and Dries, 2024). In contexts characterized by persistent price volatility, climate variability, and limited institutional protection, informality provides mechanisms through which actors can sustain coordination and continuity in their everyday economic practices. This study extends this perspective by showing that such patterns of coordination are not incidental but systematically produced through socially embedded practices that link actors, resources, and decision-making processes.

A key strength of informal systems is their capacity for adaptive coordination. Unlike formal market arrangements that rely on standardized rules and contractual enforcement, informal systems in Enrekang operate through flexible and relationally embedded practices. Farmers diversify their crops, use coffee as a reserve asset, and rely on trusted traders for input provision and market access. Traders, in turn, adjust their trading routes, source products from multiple locations, and continuously monitor market prices. These practices reflect a form of distributed adaptation in which resilience emerges through continuous adjustment rather than planned intervention (Boillat and Speranza, 2023; Folke, 2006). This is enabled by dense social networks and repeated interactions that support responsiveness in the face of uncertainty (Rouillé et al., 2024; Song, 2024). In this sense, adaptation is not organized through formal planning but emerges from ongoing interactions among actors within a system. These coordinated practices, as demonstrated in the integrated empirical findings, show that adaptation is enacted through socially embedded processes rather than discrete livelihood assets.

Simultaneously, the findings demonstrate that informality does not eliminate risk but reorganizes its distribution among actors. Trust-based credit, deferred payments, and non-contractual exchanges allow economic activities to continue, but they also shift financial exposure onto specific actors, particularly traders and informal intermediaries. Farmers are partially shielded from immediate losses through relational arrangements, whereas traders absorb the greater risks associated with price fluctuations, delayed payments, and uncertain market demand. Similar patterns have been observed in other informal market settings, where risk-sharing mechanisms sustain continuity but reproduce uneven vulnerability in the absence of formal safeguards (Johnson et al., 2023; Mkhize and Cele, 2025). This highlights that resilience in informal systems is relationally organized, but its benefits and burdens are unevenly distributed across communities.

This finding contributes to ongoing debates in the livelihood resilience literature, which cautions against equating resilience with positive welfare outcomes (Boillat and Speranza, 2023; Ruben, 2024). In Enrekang, livelihood strategies oriented toward income continuity and risk spreading enable actors to remain operational under uncertainty; however, they do not necessarily reduce long-term exposure to economic or ecological risks. Instead, uncertainty becomes normalized within everyday practices, and resilience emerges as the capacity to continue functioning rather than eliminating vulnerabilities. This distinction reinforces the need to differentiate between resilience as continuity of function and resilience as a reduction of vulnerability, which do not necessarily coincide in informal livelihood systems.

The empirical findings also highlight that the adaptive capacity of informal systems is closely linked to the interaction between social capital and other livelihood capitals. Financial capital is accessed through informal credit and savings mechanisms, human capital is mobilized through labor networks and experiential learning, and physical capital, including transport infrastructure, market facilities, and telecommunication networks, enables coordination across different spatial scales. Natural capital further shapes adaptive decisions through crop selection, diversification, and environmental variability responses. These interdependencies further indicate that physical capital, including infrastructure and technologies, is not merely accessed but co-produced and maintained through socially embedded coordination processes. This aligns with the sustainable livelihood framework, which conceptualizes resilience as emerging from the interaction of multiple livelihood capitals rather than from any single asset (Ellis and Freeman, 2004; Irawan, 2023; Scoones, 1998).

Taken together, the findings suggest that informality should not simply be understood as a constraint or symptom of institutional weakness, but as a functional mode of livelihood organization that enables coordination under structural uncertainty. However, this functionality is accompanied by trade-offs, particularly in terms of uneven risk distribution and limited institutional protection. By demonstrating how coordination and vulnerability coexist, this study contributes to reframing informality as both enabling and constraining, rather than inherently resilient or deficient. Recognizing both dimensions is essential for moving beyond simplified narratives that portray informal systems as inherently resilient or vulnerable.

### 5.3 Insights from political ecology

A political ecology perspective provides a useful lens for interpreting why informal coordination persists and why livelihood resilience remains unevenly distributed within agri-food systems characterized by weak institutional protection. Rather than treating informality as a deviation from formal market systems, political ecology situates it within the broader processes of uneven development, selective state engagement, and persistent governance gaps (Assis et al., 2023; Boamah et al., 2024; Meilinda et al., 2025). This perspective builds on foundational work that emphasizes how access to resources and livelihood opportunities is shaped by power relations rather than resource availability alone (Ribot and Peluso, 2003). The findings from Enrekang illustrate that informal arrangements are not temporary or transitional but are structurally embedded within the livelihood system. Social capital–based coordination emerges in response to the limited reach of formal mechanisms for price stabilization, risk protection, and market regulation. Extension services, infrastructure programs, and regulatory frameworks exist, but their implementation remains uneven and often program-driven. Consequently, actors rely on relational networks, shared knowledge, and informal institutions to organize production and exchange daily.

From a political ecology perspective, this reliance reflects the internalization of structural uncertainty in daily livelihood practices. Farmers, traders, and other actors adapt not because risks are reduced, but because collective mechanisms for absorbing those risks are limited. This resonates with the argument that livelihood resilience in such contexts is shaped by how communities absorb and redistribute risks in the absence of comprehensive institutional safeguards (Isakson, 2025; Reyerson, 2023). In Enrekang, practices such as trust-based credit, crop diversification, and market flexibility allow actors to remain operational; however, they do not fundamentally alter the structural conditions that generate this uncertainty. In this sense, adaptation represents a socially organized response to constraints rather than a transformation of underlying vulnerabilities.

The findings also reveal how power and positionality within social networks influence resource access and opportunities. Access to price information, trading networks, and market channels is not evenly distributed across actors. Traders with wider networks and greater mobility are better positioned to respond to price fluctuations and manage market risks, whereas farmers with limited connections face greater constraints in negotiating prices and accessing alternative markets. Similarly, access to informal credit, digital information, and infrastructure is mediated by network embeddedness, reinforcing differentiated livelihood outcomes. This demonstrates that social capital, while enabling coordination, also reproduces inequalities through differential access to networks and opportunities. These patterns align with studies showing that informal markets can reproduce hierarchies through unequal access to information and coordination mechanisms (Jayaraman et al., 2024; Kopp and Sexton, 2021). The embedded nature of infrastructure and technological development further illustrates how access to material resources is mediated through social relations and power structures, rather than determined solely by their physical availability.

Importantly, these dynamics challenge the assumption that strengthening resilience necessarily reduces vulnerability. From a political ecology perspective, resilience can coexist with persistent inequality when adaptive practices function in structurally constrained environments. In Enrekang, livelihood continuity is maintained through socially embedded coordination; however, this continuity does not imply an equitable distribution of risks or benefits. Instead, resilience reflects a socially organized response to structural constraints, where actors negotiate uncertainty through existing networks rather than through formal institutional protection. This finding reinforces a key insight from political ecology: resilience outcomes must be understood in relation to power, access, and structural conditions rather than as neutral or universally beneficial processes (Boillat and Speranza, 2023; Ribot and Peluso, 2003; Tanner et al., 2015).

This perspective has implications for policies and governance. Efforts to formalize markets or strengthen institutional control may not automatically resolve underlying inequalities if they fail to address the distribution of power, access, and resources within existing systems. This aligns with a growing body of scholarship that critiques formalization as an incomplete solution when it overlooks the socially embedded nature of economic coordination (Chen and Alter, 2012; Cleaver and Whaley, 2018; Meagher, 2010). Therefore, political ecology cautions against viewing formalization as a neutral or universally beneficial intervention. Instead, it calls for greater attention to how governance interventions interact with existing informal arrangements and whether they reinforce, reshape, or marginalize the coordination mechanisms that sustain livelihoods (Cleaver, 2012; Ostrom, 2005). This suggests that policy interventions should focus not only on improving institutional capacity but also on addressing asymmetries in access to networks, information, and resources that shape livelihood opportunities for women.

Taken together, these findings suggest that informal agri-food systems should be understood as contested and differentiated spaces in which coordination, resilience, and inequality are produced simultaneously. Livelihood resilience in Enrekang is not the outcome of stable or well-protected systems but of continuous adaptation within structurally uneven conditions shaped by governance gaps and asymmetrical power. By integrating a political ecology perspective, this study extends the analysis of social capital beyond coordination processes to reveal how power and inequality shape the distribution of resilience outcomes within informal agri-food systems in the study area.

### 5.4 Rethinking informality, risk, and governance in livelihood resilience

Research on smallholder agri-food systems increasingly recognizes that livelihoods are shaped by enduring and overlapping uncertainties rather than by isolated shocks (Boillat and Speranza, 2023; Ruben, 2024). Within this debate, informality is often framed ambivalently—at the same time as a source of flexibility and as an indicator of exclusion from formal protection (Milone and Ventura, 2024; Moreno-Miranda and Dries, 2024). The findings from Enrekang complicate this dual framing by showing that informal arrangements function as everyday livelihood infrastructure. This includes the co-production of infrastructure, technologies, and market arrangements that are often treated as separate forms of capital in conventional frameworks. Building on the analytical framework of this study, these processes are structured through the interaction of relational, cognitive, and structural dimensions of social capital, which enable actors to mobilize and combine livelihood capital. These patterns of socially embedded coordination also have implications beyond local exchange dynamics, particularly in how efficiency and resource use are organized within the system.

From a bioeconomic perspective, such arrangements can be understood in terms of how social systems optimize the use of limited resources under uncertain conditions. Efficient circulation of information, trust-based coordination, and adaptive decision-making reduce systemic inefficiencies and enhance resource-use productivity. This aligns with emerging studies linking social coordination, efficiency, and sustainability in agri-food systems (Fafchamps and Minten, 2012; Menéndez-Gámiz et al., 2026; Pérez et al., 2026). In this context, the efficiency of information flow and coordination mechanisms not only improves exchange dynamics but also shapes how material resources and infrastructures are utilized and sustained across the system.

These findings further suggest that the efficiency of information flow within social networks plays a critical role in shaping the coordination outcomes. Timely access to price signals, production information, and market opportunities enables actors to adjust their decisions in real time, thereby reducing delays, uncertainty, and transaction inefficiencies (Aker and Mbiti, 2010; Fafchamps and Minten, 2012). In this sense, social capital not only facilitates interaction but also enhances the efficiency with which resources, knowledge, and goods are mobilized across the system (D. Aldrich, 2012; Song, 2024). This contributes to system-level responsiveness and productivity, particularly in contexts where formal coordination mechanisms are limited (Menéndez-Gámiz et al., 2026; Pérez et al., 2026).

However, this infrastructure does not address structural vulnerabilities. As highlighted in Sections 5.2 and 5.3, adaptive capacity within informal systems is achieved through relational coordination, shared knowledge, and network-based arrangements; however, these mechanisms do not provide institutionalized protection against risk. Instead, protection remains contingent on social ties, reputation, and positionality within networks rather than being guaranteed through formal institutional mechanisms (Barrett and Constas, 2014; Devereux and Sabates-Wheeler, 2004; Tanner et al., 2015). In empirical terms, practices such as trust-based credit, flexible marketing arrangements, and network-based access to information enable the continuity of production and exchange but do not shield actors from price volatility or unequal bargaining power. This distinction between coordination and protection is central to understanding why resilience can coexist with persistent precarity, as highlighted in the resilience literature that differentiates between the capacity to maintain function and reduce vulnerability (Boillat and Speranza, 2023; Folke, 2006). Informal systems in Enrekang effectively coordinate livelihood activities, but they do not eliminate exposure to price volatility, production risks, or unequal access to resources.

This finding contributes to ongoing debates that question the assumption that strengthening resilience necessarily leads to improved welfare outcomes for the poor population. In line with recent critiques, resilience should not be equated with reduced vulnerability, as adaptive practices may sustain continuity while leaving the underlying structural conditions unaltered (Boillat and Speranza, 2023; Ruben, 2024). Consistent with insights from the sustainable livelihood framework, this suggests that resilience emerges from the interaction of multiple livelihood capitals, but the distribution of benefits remains conditioned by access to these capitals and the social relations that govern their use (Ellis and Freeman, 2004; Scoones, 1998). In Enrekang, strategies such as diversification, trust-based credit, and flexible market coordination enable actors to remain operational; however, they also normalize uncertainty as a routine condition of livelihood practices.

These findings also challenge policy approaches that position formalization as the primary pathway to stability and development in developing countries. While formal institutions, such as extension services, infrastructure programs, and regulatory frameworks, play important roles, their effectiveness depends on how they interact with existing informal arrangements in the region. Evidence from Enrekang shows that informal systems, including trader networks, KWT groups, arisan, and locally coordinated market practices, already perform critical coordination functions in the local economy. Formal interventions that overlook this existing systems risk misalignment with everyday practices or the unintended disruption of functional arrangements. Rather than viewing formal and informal systems as mutually exclusive, the findings suggest the need to focus on the institutional interface between them (Cleaver, 2012; Ostrom, 2005) which involves understanding how formal support, such as infrastructure development, program implementation, and extension services, can reinforce, complement, or reshape existing coordination mechanisms. The development of farm roads through collective action, later supported by government funding, as well as the operation of the STA as a formal facility embedded in relational exchange practices, illustrates how such interfaces have already emerged in practice.

From this perspective, governance in informal agri-food systems should move beyond binary distinctions—formal versus informal, resilient versus vulnerable—and instead focus on how coordination, risk, and protection are differently distributed and organized within existing livelihood systems (Jayaraman et al., 2024; Kopp and Sexton, 2021). This also implies that strengthening governance requires not only institutional expansion but also addressing asymmetries in access to networks, information, and resources that shape participation in the livelihood systems. This requires shifting attention from replacing informal arrangements to strengthening their capacity while addressing the gaps in institutional protection that contribute to uneven vulnerabilities.

Overall, the findings reframe informality not as a deviation from ideal market systems but as a structurally embedded mode of livelihood shaped by persistent uncertainty and partial governance. By distinguishing between coordination and protection, this study contributes to ongoing debates by demonstrating that informal systems can sustain livelihood resilience while simultaneously reproducing structural inequalities. Understanding how coordination and protection are produced differently within these systems is essential for advancing both theoretical and policy debates on livelihood resilience in the Global South.

### 5.5 An empirically grounded framework of social capital and livelihood resilience

Building on the empirical findings and discussion, this study proposes a refined framework illustrating how the relational, cognitive, and structural dimensions of social capital interact to coordinate livelihood practices, distribute risk, and shape resilience outcomes in informal agri-food systems. Relational social capital enables trust-based transactions and credit arrangements; cognitive social capital aligns shared interpretations of risk and acceptable practices; and structural social capital provides routinized organizational settings for repeated interactions. Together, these dimensions coordinate everyday livelihood practices while simultaneously shaping risk distribution across actors. Unlike existing frameworks that position social capital as one component within a broader set of livelihood assets (Scoones, 1998), this framework conceptualizes it as a coordinating mechanism that links actors, resources, and practices across the livelihood system, rather than as a static resource at the household level. This study extends the sustainable livelihood perspective by shifting attention from asset accumulation to the relational processes through which assets are mobilized, combined, and sustained in practice (Ellis and Freeman, 2004; Scoones, 1998). While previous studies emphasize how livelihood strategies emerge from the interaction between risks and asset portfolios at the household level (Irawan, 2023), this framework further demonstrates how these processes are coordinated across multiple actors through multidimensional social capital within informal agri-food systems.

This framework highlights that livelihood resilience emerges not from isolated assets but from the coordinated functioning of socially embedded economic relations, where continuity is maintained through interaction rather than through institutional protection. Consistent with contemporary resilience scholarship, this suggests that resilience should be understood as a process of maintaining system function under uncertainty, rather than as an indicator of reduced vulnerability or improved welfare outcomes (Barrett and Constas, 2014). The framework therefore conceptualizes physical capital not as a standalone asset, but as an outcome of coordinated social processes across relational, cognitive, and structural dimensions.

**Figure 2:**
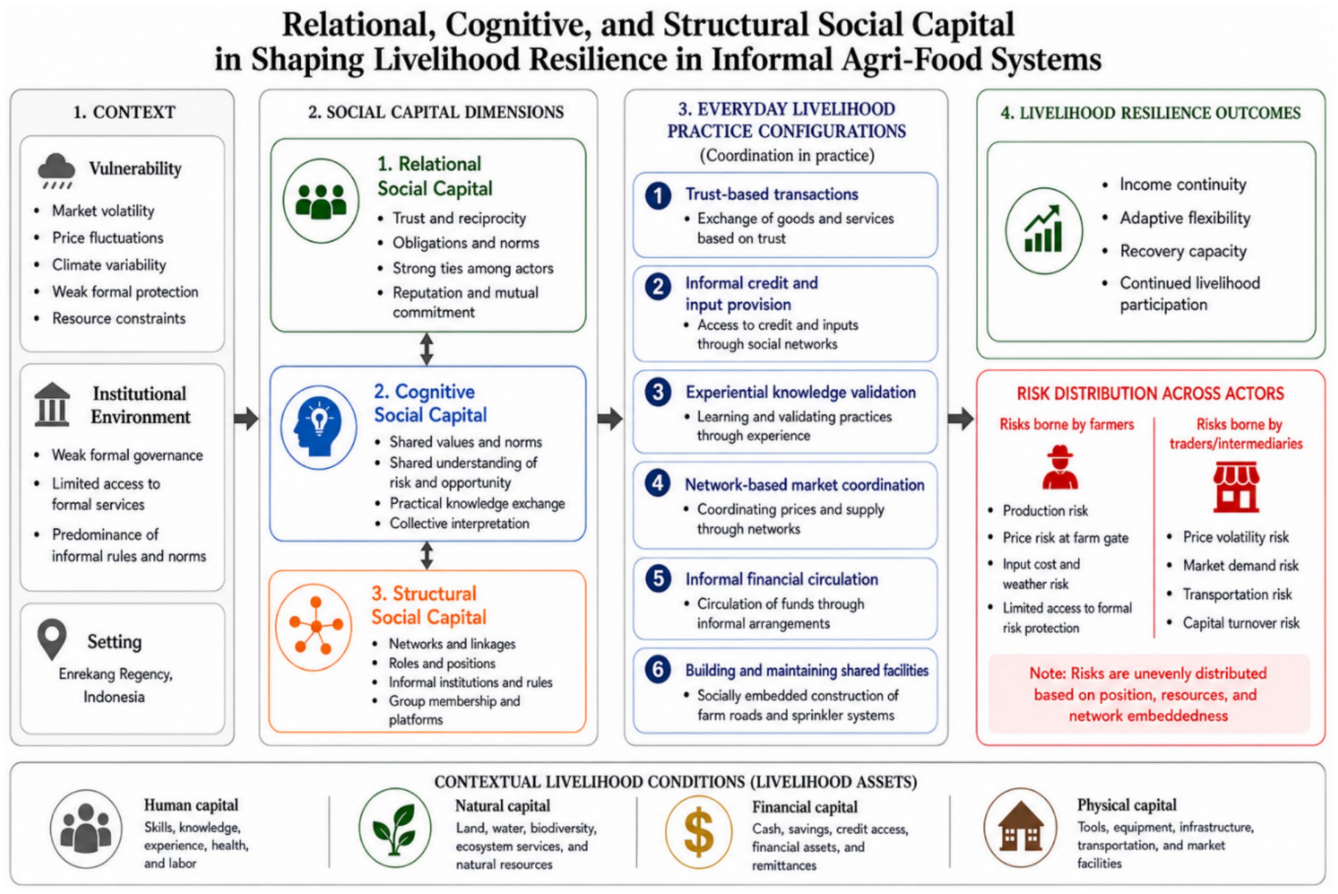
Empirically grounded framework showing how relational, cognitive, and structural social capital coordinate livelihood practices and shape resilience outcomes in informal agri-food systems, with uneven risk distribution across actors. Source: Authors’ elaboration based on empirical findings.

Figure 2 synthesizes these empirical patterns into an integrated framework derived from this study, illustrating how multidimensional social capital operates as a coordinating mechanism that shapes everyday livelihood practices within rural informal agri-food systems. The framework demonstrates how these practices generate livelihood resilience outcomes while simultaneously producing uneven risk distributions conditioned by broader livelihood contexts and resource configurations. This dual outcome—coordination alongside unequal risk exposure—represents the central contribution of the framework, highlighting that resilience in informal systems is both socially produced and experienced differently by actors.

## 6 Conclusion

This study examined how the relational, cognitive, and structural dimensions of social capital shape livelihood resilience within informal agri-food systems. Drawing on empirical evidence from Enrekang, the findings show that social capital operates not only as a supporting asset but also as a coordinating mechanism that organizes production, exchange, and adaptation across interconnected actors. Through trust-based relationships, shared interpretations, and routinized arrangements, social capital enables continuous adjustment under uncertainty.

Simultaneously, the study demonstrates that such coordination does not eliminate vulnerability but reorganizes its distribution across the actors. While farmers maintain continuity through diversification and relational support, traders and intermediaries are often more exposed to market risk. These findings highlight that livelihood resilience in informal systems is characterized by the capacity to sustain functions, rather than reducing structural vulnerability. In this sense, resilience emerges as a socially embedded and processual phenomenon shaped by ongoing interactions rather than a stable or uniformly beneficial outcome.

This study makes three contributions to the literature. First, it advances the literature on social capital by reconceptualizing it as a coordinating infrastructure that links actors, resources, and practices across livelihood systems, extending beyond its conventional treatment as an individual or community-level asset. Second, it contributes to livelihood resilience debates by clarifying the distinction between coordination and protection, showing that the ability to maintain continuity does not necessarily imply reduced exposure to risk. Third, by integrating insights from political ecology, this study demonstrates how power, access, and network embeddedness shape the uneven distribution of resilience outcomes within the informal agri-food system.

These findings have important implications for policy and practice. Interventions that focus solely on formalization or institutional expansion may overlook the critical role of existing informal arrangements in sustaining livelihoods. Rather than replacing informal systems, policy efforts should aim to strengthen their coordination capacities while addressing gaps in institutional protection, particularly regarding market risks, access to information and financial security. This includes recognizing and supporting hybrid governance arrangements, where formal and informal systems interact, such as community-based infrastructure development and locally embedded market institutions.

This study had several limitations that should be considered. The findings are based on a single case study in a horticulture-based system, which may limit its generalizability to other contexts with different commodity structures and institutional environments. In addition, while this study captures multiple actor perspectives, further research could benefit from longitudinal approaches to better understand how coordination mechanisms and risk distribution evolve. Future studies could explore comparative cases to examine how variations in institutional support and market integration influence the role of social capital in shaping livelihood resilience.

In conclusion, this study shows that informal agri-food systems are not residual or transitional but are structurally embedded modes of livelihood. This confirms that social capital functions as a coordinating mechanism through which economic practices are organized and risks are distributed across the actors. This highlights the role of efficient information flow in sustaining adaptive coordination under uncertainty. Understanding this dual role is critical for advancing both theory and policy. Moving beyond the binary distinctions between formal and informal systems, future research and interventions should focus on how these processes can be strengthened while addressing gaps in institutional protection. Advancing more equitable and resilient livelihood systems, therefore, requires strengthening these mechanisms alongside institutional protection.

## Data Availability

The datasets presented in this article are not readily available because the datasets presented in this article consist of qualitative interview transcripts and field notes. Due to the need to protect participant confidentiality, the data are not publicly available. De-identified data may be made available by the corresponding author upon reasonable request and subject to ethical approval. Requests to access the datasets should be directed to darsalman@agri.unhas.ac.id.
